# Smoking cessation programmes for women living in disadvantaged communities, “We Can Quit 2”: A systematic review protocol

**DOI:** 10.12688/hrbopenres.12901.1

**Published:** 2019-06-12

**Authors:** Emma Burke, Fiona Dobbie, Nadine Dougall, Mary Adebolu Oluwaseun, David Mockler, Joanne Vance, Nicola O'Connell, Catherine Darker, Linda Bauld, Catherine Hayes

**Affiliations:** 1 School of Medicine, Trinity College Dublin, Dublin, Ireland, D24 DH74, Ireland; 2 Usher Institute, College of Population Health Sciences Medicine and Informatics Veterinary Medicine, University of Edinburgh, Teviot Place, Edinburgh, EH8 9AG, UK; 3 School of Health and Social Care, Edinburgh Napier University, Sighthill Court, Edinburgh, EH11 4BN, UK; 4 Irish Cancer Society, Dublin, DO4 VX65, Ireland

**Keywords:** Smoking Cessation, Women and Smoking, Community-based Intervention, Social and Health Inequalities, Cluster Randomised Controlled Trial, Feasibility study, Pilot trial, Systematic Review.

## Abstract

Tobacco use is the leading cause of preventable death in Ireland with almost 6,000 smokers dying each year from smoking-related diseases. Amongst younger Irish women, smoking rates are considerably higher in those from socially disadvantaged areas compared to women from affluent areas. Women from poorer areas also experience higher rates of lung cancer. To our knowledge, there are no peer reviewed published systematic reviews on the effectiveness of interventions tailored to reduce smoking rates in women from disadvantaged areas. This systematic review protocol will aim to examine the effectiveness of such interventions and to describe trial processes such as recruitment, follow-up and dropout prevention strategies, as well as barriers and enablers of successful implementation.

A systematic review will be conducted of peer-reviewed randomised controlled trials and associated process evaluations of smoking cessation interventions designed for women living in socially disadvantaged areas. If the search returns, less than five studies are review criteria will expand to include quasi-experimental studies. A number of databases of scholarly literature will be searched from inception using a detailed search strategy. Two independent reviewers will screen titles, abstracts and full-text articles to identify relevant studies using a pre-defined checklist based on PICOS. In the case of disagreement, a third reviewer will be consulted. The quality of included studies will be assessed using the ‘Grading of Recommendations Assessment, Development and Evaluation’ (GRADE) criteria. Quantitative data will be extracted and, if comparable, will be assessed using meta-analysis. A narrative meta-synthesis of qualitative data will be conducted.

This review aims to synthesise information from relevant studies on smoking cessation interventions tailored for women from socially disadvantaged areas. The evidence obtained from studies and presented in this review will help guide future research in this area.

**Registration: **This review will be registered with International Prospective Register of Systematic Reviews (PROSPERO).

## Introduction

Tobacco smoking remains the leading cause of morbidity and mortality globally. According to a recent World Health Organisation (WHO) report, tobacco kills more than 7 million people each year ^1^. More than 6 million of those deaths are the direct result of tobacco use, with 890,000 caused by non-smokers’ exposure to second-hand smoke. Compared to the rest of the world, the WHO European Region has one of the highest proportions of deaths attributable to tobacco use. WHO estimates that tobacco use is responsible for 16% of all deaths in adults over 30 years in the European Region, with many of these occurring prematurely ^2^.

The Healthy Ireland Survey 2018 reported 20% of people aged 15 years are current smokers; with a rate of 22% in men and 17% in women ^3^. Cigarette smoking prevalence were highest among 25 to 34-year olds, with rates declining with age ^3^.

While tobacco use was previously largely a male phenomenon, the gap in prevalence between genders is now less than 5% in countries such as Denmark, the Netherlands, Sweden and the United Kingdom ^4^. However, research has highlighted that gender remains an important factor when considering smoking prevalence in areas of deprivation. A recent report by Health Ireland Survey (2018) ^3^ found that smoking rates were higher in more disadvantaged areas than in affluent areas (26% versus 16%, respectively). Women aged 18 to 29 from the lowest socioeconomic groups in Ireland have been reported to have almost double the smoking rate compared to those belonging to affluent groups (56% versus 28%) ^5^. This disparity was also observed in men, although the difference was less pronounced (44% versus 31%) ^5^.

Smoking perpetuates health inequalities between women from richer and poorer communities. Data from 2016 found significantly higher lung cancer rates in women from the most deprived areas of Ireland versus women from the least deprived (age standardised rate ratio, 1.56; 95% CI, 1.42 1.72) ^6^. Lung cancer incidence in Irish men has declined in the period from 1994 to 2015, but has increased in women over the same period ^7^. More women in Ireland are now dying from lung cancer than breast cancer ^8^. Smoking can have differential effects on women’s health such as increased risk of cervical cancer, breast cancer and premature menopause ^9^, and there may be a cumulative effect of disadvantage on women who smoke and their health outcomes. These results suggest female smokers from more disadvantaged groups should be targeted for greater support in smoking cessation, and there have been recent calls for the development of tailored interventions in this area ^10^.

In a previous systematic review of randomised controlled trials (RCTs) of smoking trials, women had more difficulty in maintaining long-term abstinence compared to men ^11^. A general population survey across several countries found that amongst people aged under-50, women were more likely to stop smoking completely compared to men, but amongst older groups, men were more likely to quit than women ^12^. It is likely deprivation and gender interact to accentuate these differences and there is a need to expand and explore the scope of such findings.

To our knowledge there are no peer reviewed, published systematic reviews that examine the smoking cessation interventions targeted at women living in disadvantaged areas. A previous narrative review assessed gender differences in smoking cessation ^11^ but did not specifically explore the role of disadvantage. This systematic review will evaluate the effective of smoking cessation interventions tailored to women living in disadvantaged areas.

### Aims and research objectives

This systematic review protocol sets out to identify, appraise and synthesise the existing evidence of effectiveness of smoking cessation programmes available to women living in disadvantaged communities. 

The review will address the following research objectives:

1. Assess the effectiveness of intervention programmes for smoking cessation in women living in disadvantaged communities;
2. Identify the recruitment strategies used by these programmes and quantify their success in the recruitment of participants;
3. Identify the retention, drop-out and follow-up rates of the programmes;
4. Identify the implementation strategies used by these programmes, e.g. training, coaching.
5. Identify the barriers and enablers to successful implementation of the programmes.

## Methods

A systematic review of peer-reviewed literature will be conducted on smoking cessation and quit-tobacco interventions for women living in socially deprived areas. The proposed review will be guided by the “Preferred Reporting Items for Systematic Review and Meta-Analysis Protocols” (PRISMA-P) checklist ^13^; a completed PRISMA-P checklist is available ^14^. This review protocol will be registered with PROSPERO (awaiting registration number).

### Study design

The systematic review will consider RCTs and associated process evaluations. If the search returns fewer than five studies, review criteria will be expanded to include quasi-experimental studies of smoking cessation interventions designed for women living in socially disadvantaged areas.

### Search strategy

The strategy aims to find published articles by a systematic search of Medline, Embase, Cochrane Library of Systematic Reviews, Cinahl, PsycINFO, Web of Science, Scopus, Sociological Abstracts, ASSIA, British Nursing Index, Google Scholar, Epistemonikos, with relevant MeSH headings. An experienced librarian (D.M.) will develop a sensitive search strategy for each individual database. There will be no restriction on country or year of publication; however, all papers must be in English. Where studies are not available we will contact study authors. This study will exclude grey literature, conference abstracts, opinion pieces, literature reviews commentaries and editorials. Bibliographies of all retrieved trials and other relevant publications, including reviews and meta-analyses will be checked for additional relevant articles.

### Study eligibility

The terms of this review will be defined using PICOS (Population, Intervention, Comparison, Outcome, and Study Design).

### Population

Women aged 18 years and above that are reported as being from disadvantaged communities who smoke and who have attended any type of smoking cessation programme.

### Intervention

Intervention programmes that report smoking cessation outcomes at end of programme delivery and follow-up including any recruitment strategies used in the studies. 

‘Smoking cessation interventions’ will be defined as interventions that are designed to assist smoking cessation. These are predicted to consist of the following:

- Any RCTs that report individual–level interventions such as (i) brief advice to stop smoking from a health professional (e.g. physician); (ii) pharmacotherapy (nicotine replacement therapies such as the transdermal patch, chewing gum, nasal sprays, lozenges, inhalers, dissolvable strips, and prescribed or self-administered alternatives to tobacco such as bupropion/varenicline or e-cigarettes); or (iii) behavioural support (any form of encouragement, advice or discussion from a trained stop-smoking specialist).
- Any RCTs that report accompanying process evaluations that focus on implementation strategies, barriers and facilitators to the implementation of the intervention during the programmes.

### Comparison

Corresponding information will be extracted for the control arm of RCTs, typically ‘care as usual’.

### Outcome

The primary outcome of interest is the proportion of the population randomised who achieve smoking cessation (e.g. abstinence). Smoking cessation will be defined as an intention-to-stop smoking cigarettes from a given point in time (e.g., a ‘quit attempt at 4 weeks’), followed by resistance of urges to smoke, resulting in a period of abstinence ^15^, that will be corroborated by a saliva test. 

Secondary outcomes will be proportions of the population i) recruited from those eligible; ii) retained at the end of the intervention; and iii) retained at subsequent follow-up data collection points.

### Study design

***Inclusion criteria***

- RCTs trials of women aged 18 years and above who smoke and have attended any type of smoking cessation programme and live in disadvantaged communities.
- RCTs with both men and women in the same sample, if all findings were reported separately for women.
- RCTs with a sample of pregnant and non-pregnant women, if smoking cessation is reported separately for non-pregnant women.
- RCTs that include women living in any circumstances, but the results are segmented into smoking cessation for women in disadvantaged communities.
- Any definition of “Disadvantaged communities” (including but not limited to poverty, low income, unemployment, educational status, social class, social condition, neighbourhood/area status) ^16^.

***Exclusion criteria.*** This review will exclude studies that are exclusive to pregnant women.

### Data selection and management

The title and abstracts retrieved from the electronic databases and references will be exported to EndNote bibliographic software for storage and the removal of duplicates. After removal of duplicates, the title and abstracts will be exported to Covidence software for reviewing ^17^. Two independent reviewers will identify relevant titles and abstracts using a pre-defined checklist based on PICOS. If the reviewers deviate in their judgement, a third reviewer will assess these abstracts. The full text version will be obtained for the remaining relevant searches. The two independent reviewers will review the full text and those deemed irrelevant will be removed. Any disagreements that arise between the two reviewers will be resolved through discussion with a third reviewer ^13^.

### Quality assessment for risk of bias

Two reviewers will independently check each selected article to minimise bias. All selected articles will be judged for their quality based on the Grading of Recommendations Assessment, Development and Evaluation’ (GRADE) system ^18^. The GRADE approach provides guidance on rating the quality of research evidence in health care. This system addresses the five main factors that can downgrade the quality scores of RCTs ^18^, which include risk of bias, inconsistency, indirectness, imprecision and publication bias. Evidence from non-randomised studies begins as low-quality evidence, but ratings can be upgraded (provided no other limitations have been identified according to the five factors). Upgrading occurs if there is a large magnitude of effect, evidence of a dose-response effect, and all plausible confounding factors have been taken into account ^19^. An advantage of GRADE is that it leads to more transparent judgements about the quality of evidence and can help indicate the strength of recommendations based on the evidence ^20^. The GRADE approach is comprehensively described in an online manual ^19^ (freely available for download with the GRADEpro software).

### Data extraction

Using standardized data extraction forms, two reviewers will extract data from valid and selected papers for data analysis. The data extracted will include details specific to the review’s primary and secondary outcomes and fulfils the requirements for the both the outcomes and a potential meta-analysis. All corresponding authors will be contacted for key information when data are ambiguous or missing from the published study. Data extraction will be independently crosschecked, reviewers will resolve disagreements by discussion, and a third reviewer will resolve unresolved disagreements.

### Data synthesis

For quantitative data, where possible, odds ratios for binary outcome data and their 95% confidence intervals will be calculated from data generated by each included RCT. Data derived via intention-to-treat analysis will be used. If possible, results from comparable groups of studies will be pooled into statistical meta-analysis using Review Manager software from the Cochrane Collaboration ^21^.

Statistical heterogeneity between combined studies will be tested using the *I ^2^* method alongside the standard chi-square test. An estimate greater than or equal to 50% accompanied by a statistically significant Chi ^2^ statistic will be interpreted as evidence of statistical heterogeneity ^22^. If substantial levels of heterogeneity are found for the primary outcome measure, all data entered will be checked for accuracy, then a visual inspection of the data will take place and removal of outlying studies will be carried out to assess if heterogeneity persists. Sub-group analyses will be completed if sufficient data are available to examine between-study variability on, for example categories of intervention, or risk of bias.

The unit of analysis is expected to be at an individual level outcome. Where ‘cluster randomisation’ is used, these data will be extracted alongside an assessment of whether the authors accounted for intra-class correlation (ICC) in clustered studies. Where clusters have not been incorporated into the analysis of the trial, authors will be contacted and asked to provide the ICCs for their clustered data. These binary data derived from randomised cluster trials will then be divided by a ‘design effect’, estimated using the mean number of participants per cluster (m) and the ICC using the formula (Design effect = 1 + (m−1) × ICC] ^23^). If the ICC cannot be obtained, we will assume it to be 0.1 ^24^.

For any particular outcome, if more than 50% of data were unaccounted for, we will not use these in any meta-analysis. A random effects model will be used in preference to a fixed-effects model to combine individual RCT primary outcome measures as it takes into account that different trials are estimating different but related intervention effects.

Where statistical pooling is not possible the findings will be presented in narrative form. Heterogeneous qualitative data will be synthesised in a narrative format focused around the review’s objectives with findings presented thematically; however, where possible, meta-analysis will be carried out on homogenous data.

## Discussion

This review will systematically examine the available evidence of effectiveness on smoking cessation interventions in women from socially disadvantaged areas. By summarising recruitment implementation strategies and barriers. It is hoped that these findings will help direct future research and smoking cessation policy. This review will focus on RCTs and associated process evaluations. If the search returns fewer than five studies, the review criteria will expand to include quasi-experimental studies. There is no restriction to the country of origin or year of publication, however all language must be in English. The aim of this review RCT is to help guide future smoking cessation programmes for women from disadvantaged areas.

## Limitations

There are some limitations to the outlined systematic review. The restriction to English is acknowledged as a language bias. The cost of high-quality translations of in-depth qualitative data are beyond the resources of this review; however, non-English language studies identified at the screening stages and excluded from the synthesis will be listed in an appendix to the review to aid future reviewers.

## Data availability

### Underlying data

No underlying data are associated with this article.

### Reporting guidelines

Open Science Framework: PRISMA-P checklist for “Smoking cessation programmes for women living in disadvantaged communities, “We Can Quit”: A systematic review protocol”. https://doi.org/10.17605/OSF.IO/J96M5 ^14^.
